# Different whole body HIIT protocols do not promote different muscle thickness and functional adaptations among healthy physically active subjects

**DOI:** 10.3389/fspor.2024.1513030

**Published:** 2025-01-15

**Authors:** Alexandre L. Evangelista, Júlio B. B. de Camargo, Roberta L. Rica, Luiz Carlos Carnevali Júnior, Gregg S. Mallett, Valentina Bullo, Marco Bergamin, Stefano Gobbo, Danilo S. Bocalini

**Affiliations:** ^1^ Department of Physical Education, Italo Brasileiro Catholic College, São Paulo, Brazil; ^2^ Department of Physical Education and Motricity, Federal University of São Carlos, São Paulo, Brazil; ^3^ Department of Physical Education, Estacio de Sá Universitary Center, Vitoria, Brazil; ^4^ Department of Physical Education, Phorte College, São Paulo, Brazil; ^5^ Department of Kinesiology, Health Promotion, and Recreation, University of North Texas, Denton, TX, United States; ^6^ Department of Medicine, University of Padova, Padova, Italy; ^7^ Center of Physical Education and Sport, Federal University of Espirito Santo, Vitoria, Brazil

**Keywords:** high-intensity interval training, muscle thickness, cardiorespiratory fitness, muscle fitness, exercise protocols

## Abstract

**Introduction:**

Despite robust evidence on the benefits of high intensity interval training using body weight (WB-HIIT), the effects of different training configurations on morpho-functional adaptations are still unclear. Therefore, the aim of the present study was to assess the effects of two distinct WB-HIIT protocols on morphological and general fitness adaptations in healthy active young individuals.

**Methods:**

Thirty-four participants (22 males and 12 females) were randomly assigned to one of the following groups: 30 s of all-out effort interspersed with 10 s of passive recovery (G30 × 10, *n* = 17) or 40 s of an all-out effort interspersed with 20 s of passive recovery (G40 × 20, *n* = 17). Nine exercises were performed for both protocols, in two weekly sessions, during a 6-week intervention period. Morphological (ultrasound-derived muscle thickness of the vastus lateralis [MTVL]) and general fitness (muscle endurance and maximal oxygen consumption) assessments were performed at pre- and post-intervention moments.

**Results:**

Both training protocols elicited significant improvements in all dependent variables (*p* < 0.05), with no significant between-group differences.

**Conclusion:**

Regardless of the training configuration, both WB-HIIT programs serve as time-efficient strategies to induce changes in muscle thickness of the vastus lateralis and functional adaptations in healthy, physically active young individuals.

## Introduction

High-intensity interval training using body weight (WB-HIIT) has gained prominence as an effective and accessible strategy for improving physical fitness, health, and quality of life (1, 2). Importantly, WB-HIIT has been shown to induce similar cardiorespiratory [i.e.,; maximal oxygen consumption (VO2max); cardiac autonomic function] (3) and neuromuscular adaptations (3–5) compared with traditional cycling and treadmill-based HIIT and Moderate-Intensity Continuous Training (MICT) in healthy adults. These effects of WB-HIIT on cardiorespiratory fitness may be attributed to its all-out effort nature (above 100% VO_2_max), which would significantly elicit both central (e.g., increased cardiac output) and peripheral adaptations (e.g., enzymatic adaptations or increased mitochondrial volume and density) as well (6). For neuromuscular responses, both strength and morphological adaptations previously reported to be induced by WB-HIIT programs (7) could be mainly attributed to the fact that WB-HIIT involves high execution speed and stretch-shortening cycles that favor recruitment of type II muscle.

Other advantages of WB-HIIT include its efficiency in terms of time (8), the possibility of performing this exercise in different environments, without the need for specific equipment (9), and a higher self-efficacy and greater enjoyment compared to traditional training approaches (10). Altogether, these characteristics are important when considering individual preferences related to gym membership, the costs involved, and the training environment (11).

However, few studies directly compared the effects of different WB-HIIT configurations on morphofunctional adaptations. Moghaddam et al. (12), for example, demonstrated similar skeletal muscle cross sectional areal accrual following two different WB-HIIT protocols (10-5-HIIT or 20-10-HIIT) after 4 weeks. In a latter study from the same research group (13), both protocols were also equally able to induce significant increases in VO_2_max. Therefore, given the scarce nature of the literature comparing distinct WB-HIIT programs and the need for a better understanding whether these different training regimens could somehow maximize the benefits to health and physical performance, the present study aimed to verify whether different WB-HIIT protocols could generate different muscle thickness and functional adaptations in healthy, physically active individuals. Our initial hypothesis was that both groups would present improvements in morphological variables in a similar way, regardless of the training configuration.

## Methods

### Participants

Thirty-four healthy subjects (men *n* = 22 and women *n* = 12), volunteered to participate in the study. The process of recruiting volunteers for the study was conducted among Physical Education students during the academic semester. Participants were selected by distributing informational flyers and sharing announcements in classrooms. The materials contained details about the purpose of the study, eligibility criteria, procedures involved, and potential benefits of participating. Interested students were encouraged to sign up and participate voluntarily.

To be able to participate, participants were required not to present cardiometabolic conditions or medication usage that could interfere in the outcomes being assessed. Participants reported performing regular exercise for at least 150 min/week, predominantly running, cycling, fitness training, and ball sports (International Physical Activity Questionnaire-IPAQ) (14) according to the recommendations of the World Health Organization (15). After approval from the Research Ethics Committee (no. CAAE 41375120.6.0000.5542), participants read and signed an informed consent document. The study was carried out according to the Declaration of Helsinki.

### Experimental procedures

The experimental period lasted 8 weeks: the first week was a familiarization period and pre-intervention tests (baseline); the second to seventh weeks were training intervention periods; and the eighth week was post-intervention tests. Assessments of muscle thickness (primary outcome) and aerobic fitness and general fitness (secondary outcomes) were performed in pre- and post-intervention moments.

Two testing days were adopted for both assessment moments, separated by at least 72 h. The first visit consisted of muscle thickness and aerobic fitness assessments. The volunteers were also weighed and measured for height. On the second visit, general fitness tests were performed. After baseline measurements, participants were randomly assigned, by simple draw, to one of the WB-HIIT groups: a group performing 30″ of all-out effort followed by 10″ of passive rest (G30 × 10; *n* = 17) or a group performing 40″ of all-out effort followed by 20″ of passive rest (G40 × 20; *n* = 17). Both groups displayed with 11 men and 6 women.

Participants were instructed to refrain from intense exercise and alcohol for 72 h before measurements. All assessments were performed by the same researchers. No restricted dietary control was adopted, but the subjects were instructed by a nutritionist not to change their dietary intake/usual nutrition during the entire study period. Both groups also received general guidance on healthy eating habits at the beginning of the study and were allowed to continue with their regular physical activity during the study period.

### Muscle thickness

Ultrasonography was used to determine the muscle thickness of the vastus lateralis (MTVL), using an ultrasound-imaging unit (Mindray; DP10; Shenzhen, China), with a wave frequency of 7.5–10 megahertz (MHz). The ultrasound probe was applied perpendicularly to the skin for measurement. A water-soluble gel was used on the transducer to aid acoustic coupling and remove the need for excess contact pressure on the skin. Muscle thickness was defined as the distance between the interface of the muscle tissue and subcutaneous fat to the bones. Imaging was performed on the right side of the subjects' body. The subjects were asked to fast for 3 h before the tests, and muscle thickness (MT) assessments were performed at the same time of day at pre- and pos*t*-testing.

The assessments were performed at 50% distal between the lateral condyle of the femur and greater trochanter (16) with the subjects resting supine on an examination bed, with their knees fully extended and relaxed. The examined limb was secured to minimize undesired movements. The same researcher was responsible for carrying out both evaluations and was blinded to the intervention group of the participants during the experimental period. The values of the coefficient of variation and standard error of measurement were 3.18% and 0.76 mm, respectively (data from our laboratory).

### General fitness measures

Muscular endurance was evaluated by the sit-up, push-up, and Burpee's squat thrust tests. The sit-up test was conducted with participants initially positioned on a mat (supine position), with their feet fixed on the ground, heels together, and at 30–45 cm from the hip, with the fingers intertwined behind the head. The elbows were required to touch the knees at the anterior flexion of the spine and each repetition was counted as the subject returned to the initial position. Maximum repetitions performed correctly within 1 min were recorded (17).

For the push-up test, participants were instructed to initiate the test in a prone position (facing downward) with their hands placed on the floor, slightly wider than shoulder-width apart, and the feet (also placed on the floor) either together or shoulder-width apart, ensuring the body is straight from head to heels, forming a plank-like posture. In addition, the elbows were completely extended and the trunk away from the floor. In the descendent phase, the upper body needed to touch the floor, and the hands should be lifted for one second to ensure the body is completely flat on the floor. One repetition was counted when the body moved back to the starting position and the maximum number of repetitions completed was recorded (2). When the participant was unable to raise their elongated torso and lower body from the floor, the test was finished.

For the Burpee's squat thrust tests [adapted from Vandana et al. (18)], participants started in a standing position and were instructed to squat down and kick out their legs. The participants were then instructed to perform the reverse order of movements, to complete one full repetition (19). From the signal “go” the participant was asked to repeat this movement as rapidly as possible and the number of movements completed was recorded. When participants were unable to perform the movement properly, the test was finished. A 10 min rest interval was adopted between tests. All tests were performed in the same order during both pre- and post-intervention assessments. In addition, all researchers that carried out the assessments were blinded to the intervention group of the participants during the experimental period.

### Maximum oxygen consumption

The maximum oxygen consumption (VO_2_max) was measured using the Yo-Yo Endurance test. In summary, all participants were lined up along the starting line with one foot behind the line (cone A) and began running when instructed by the audio recording, when signaled to by the recorded audio beep (cone B), the volunteers turned and returned to the starting point. The participant continued to shuttle back and forth between the two lines 20 m apart, in time to the audio cues. At regular intervals, the time between audio signals was reduced and the running speed needed to be increased. The test was finished when the participants were not able to follow the beep two times in a row (20, 21). The distance (meters) covered in the test was then recorded and, in order to estimate the VO_2_max, the following equation was adopted: VO_2_max (ml.kg.min^−1^) = (Distance covered in meters * 0.0084) + 36.4. The same researcher was responsible for carrying out both evaluations and was blinded to the intervention group that the participants were allocated to during the experimental period.

### Training intervention

The G40 × 20 WB-HIIT training session involved 1 min of general warm-up (stationary running) followed by 9 exercises (40 s stimulus in *all-out* intensity of effort) divided into 3 blocks with 2 sets each. Passive rest intervals of 20 s between exercises, 40 s between sets, and 60 s between blocks were given (Table 1). The exercises adopted were, Block 1: Squat Jump, Curl up, and Skipping; Block 2: Wide arms push-up, “sumo” squat, and mountain climber; Block 3: Jumping jack, push-up, and spider plank.

**Table 1:** Description of G40 × 20 whole-body high-intensity interval training protocol.

Block 1	Set 1	Squat Jump	20 s rest	Curl up	20 s rest	Skipping	40 s rest
	Set 2	Squat Jump		Curl up		Skipping	60 s rest
Block 2	Set 1	Wide arms push-up	20 s rest	“Sumo” squat	20 s rest	Mountain climber	40 s rest
	Set 2	Wide arms push-up		“Sumo” squat		Mountain climber	60 s rest
Block 3	Set 1	Jumping jack	20 s rest	Push-up	20 s rest	Spider plank	40 s rest
	Set 2	Jumping jack		Push-up		Spider plank	60 s rest

The G30 × 10 WB-HIIT training session involved 1 min of general warm-up (stationary running), followed by 9 exercises (30 s stimulus in *all out* intensity) divided into 3 blocks with 3 sets each. Passive rest intervals of 10 s between exercises, 30 s between sets, and 60 s between blocks were allowed (Table 2). All exercises were the same as adopted in the G40 × 20 protocol. All sessions for both protocols were supervised by researchers not involved in data analysis.

**Table 2:** Description of G30 × 10 whole-body high-intensity interval training protocol.

Block 1	Set 1	Squat Jump	10 s rest	Curl up	10 s rest	Skipping	30 s rest
	Set 2	Squat Jump		Curl up		Skipping	30 s rest
	Set 3	Squat Jump		Curl up		Skipping	60 s rest
Block 2	Set 1	Wide arms push-up	10 s rest	“Sumo” squat	10 s rest	Mountain climber	30 s rest
	Set 2	Wide arms push-up		“Sumo” squat		Mountain climber	30 s rest
	Set 3	Wide arms push-up		“Sumo” squat		Mountain climber	60 s rest
Block 3	Set 1	Jumping jack	10 s rest	Push-up	10 s rest	Spider plank	30 s rest
	Set 2	Jumping jack		Push-up		Spider plank	30 s rest
	Set 3	Jumping jack		Push-up		Spider plank	60 s rest

The training routine lasted 6 weeks, since this time frame has been previously shown to induce relevant adaptations in both cardiorespiratory and morphological outcomes (22–24). Two weekly sessions were performed throughout the intervention period, since previous evidence suggest this training frequency as suitable for both beginner and intermediate individuals (11). Whenever a volunteer was absent, the training session was performed on another day in the same week. As a result, the adherence rate was 100% for both protocols.

### Statistical analysis

The normality and homogeneity of variance were analyzed using the Shapiro-Wilk and Levene tests, respectively. The mean, standard deviation (SD), and 95% confidence interval (95% CI) were calculated for each dependent variable. To compare between-groups baseline characteristics, an unpaired student *t*-test was adopted. A repeated measures analysis of variance (ANOVA) was used to compare the effects of time (pre vs. post) and groups (G30 × 10 and G40 × 20), as well as the group vs. time interaction for the variables MTVL, Sit-up, Push-up, Burpee's squat thrust, and VO_2_max. In case of significant F values, a Bonferroni *post-hoc* test was used for paired comparisons. The assumptions of sphericity were assessed using the Mauchly test. When violated, the Greenhouse-Geisser correction factor was applied. If any participant presented a pre-post change above 3 SD (outlier), his/her data was removed from the analysis. The effect size between groups (and the respective 95% CI) was calculated using Cohen's *d* and interpreted qualitatively as follows: trivial (<0.2), small (0.2–0.5), moderate (0.6–1.2), large (1.2–2.0), and very large (>2.0). A *priori* sample size calculation was performed considering fat free mass as the outcome measure, with a power of 0.80 and a target effect size of 0.38 (7), which required a minimum of 16 participants in each group to be included. The significance value adopted was *p* ≤ 0.05. All analyses were conducted in SPSS version 21 (IBM Corp, Armonk, NY).

## Results

No significant differences from baseline were noted for any of the anthropometric variables in each group (all *p* > 0.05, Table 3). Thirty-four participants completed the intervention period, and the adherence for the intervention was 100% for both groups. One subject from the G30 × 10 group did not attend the final Yo-Yo testing session. Therefore, data from this participant were not considered for the analysis of this variable. Table 4 displays the pre- and post-intervention values for all the dependent variables assessed for each experimental group.

**Table 3:** Baseline values for anthropometric variables for each experimental group.

Variables	G30 × 10	G40 × 20	*p*-value
Age (years)	23.8 ± 8.7	25.5 ± 6.8	0.517
Weight (kg)	71.0 ± 17.1	73.1 ± 11.3	0.683
Height (cm)	172.1 ± 9.1	169.7 ± 5.8	0.376
BMI (kg/m^2^)	23.9 ± 4.9	26.2 ± 5.6	0.201

Mean ± standard deviation.

kg, kilograms; cm, centimeters; BMI, body mass index.

**Table 4:** Pre- and post-intervention values for the dependent variables assessed for each group (mean ± SD).

Variables	Pre	Post		MD (95%CI)	ES
			*Δ*%		*d*
MTVL (mm)					
G30 × 10	22.8 ± 5.6	24.6 ± 5.2^a^	7.8	1.8 (0.9–2.7)	0.08
G40 × 20	23.4 ± 3.8	26.3 ± 4.0^a^	12.3	2.9 (2.2–3.5)	0.18
Sit-up (reps)					
G30 × 10	29.5 ± 8.4	32.8 ± 9.3^a^	11.1	3.4 (1.4–5.2)	0.10
G40 × 20	33.3 ± 9.6	38.2 ± 8.0^a^	14.7	4.9 (1.8–8.1)	0.13
Push-up (reps)					
G30 × 10	28.5 ± 11.9	33.6 ± 10.7^a^	17.8	5.1 (0.7–9.4)	0.11
G40 × 20	35.4 ± 16.1	41.2 ± 18.5^a^	16.3	5.9 (1.0–10.8)	0.09
Burpee's squat thrust (reps)					
G30 × 10	29.9 ± 17.1	42.3 ± 27.8^a^	41.4	12.4 (3.3–21.4)	0.17
G40 × 20	29.1 ± 21.4	43.5 ± 26.5^a^	49.4	14.4 (6.6–22.3)	0.16
VO_2_max (ml.kg.min^−1^)					
G30 × 10	41.3 ± 2.6	45.1 ± 4.3^a^	9.2	3.8 (2.7–4.9)	0.35
G40 × 20	40.9 ± 2.2	45.0 ± 3.8^a^	10	4.1 (2.8–5.4)	0.44

MTVL, Muscle Thickness of the Vastus Lateralis; reps, number of repetitions; VO_2_max, maximal oxygen consumption during Yo-Yo test; MD, mean difference; CI, confidence interval; ES, effect size; *d*, Cohen's d.

^a^ Significantly different from baseline.

### Muscle thickness

For MTVL, a significant main effect of time (*F*_1,16_ = 65.645; *p* = 0.001), but no effect of group (*F*_1,16_ = 0.638; *p* = 0.436) or group × time interaction (*F*_1,16_ = 2.750; *p* = 0.117) was observed (Table 4). No significant difference was noted for the absolute increase from baseline between groups (mean difference = 1.01 ± 0.58 mm, 95% CI = −0.18 to 2.20 mm). For the between-group ES comparison, a trivial effect was observed (*d* = −0.13). Twelve (70.5%) and sixteen (94.1%) participants from the G30 × 10 and G40 × 20 (respectively) responded above the typical error of measurement.

### General fitness measures

For the sit-up test, a significant main effect of time (*F*_1,16_ = 23.000; *p* = 0.001), but no effect of group (*F*_1,16_ = 2.323; *p* = 0.147) or group × time interaction (*F*_1,16_ = 0.617; *p* = 0.444) was observed (Table 4). No significant difference was noted for the absolute increase from baseline between groups (mean difference = 1.58 ± 1.88 repetitions, 95% CI = −2.24 to 5.42 repetitions). For the between-group ES comparison, a trivial effect was observed (*d* = −0.09).

A significant main effect of time (*F*_1,16_ = 10.798; *p* = 0.005), but no effect of group (*F*_1,16_ = 3.105; *p* = 0.097) or group × time interaction (*F*_1,16_ = 0.053; *p* = 0.821) was observed for the push-up test (Table 4). No significant difference was noted for the absolute increase from baseline between groups (mean difference = 0.7647 ± 3.336 repetitions, 95% CI = −6.03 to 7.56 repetitions). For the between-group ES comparison, a trivial effect was observed (*d* = −0.02).

Similar results were noted for the burpee's squat thrust, where a significant main effect of time (*F*_1,16_ = 22.676; *p* = 0.001), but no effect of group (*F*_1,16_ = 0.001; *p* = 0.980) or group × time interaction (*F*_1,16_ = 0.093; *p* = 0.764) was observed (Table 4). After detecting outliers (individual responses above 3 SD), one subject from each group was removed, and no significant difference was noted for the absolute increase from baseline between groups (mean difference = 2.813 ± 3.314 repetitions, 95% CI = −6.031 to 7.560 repetitions). For the between-group ES comparison, a trivial effect was observed (*d* = −0.03).

### Maximal oxygen consumption

A significant main effect of time (*F*_1,15_ = 57.758; *p* = 0.001), but no effect of group (*F*_1,15_ = 0.155; *p* = 0.709) or group × time interaction (*F*_1,15_ = 0.287; *p* = 0.600) was observed for VO_2_max (Table 4). After detecting outliers (individual responses above 3 SD), one subject from the G40 × 20 group was removed, and no significant difference was noted for the absolute increase from baseline between groups (mean difference = 0.06 ± 0.78 ml/kg/min, 95% CI = −1.56 to 1.69 ml.kg.min^−1^). For the between-group ES comparison, a trivial effect was observed (*d* = −0.03).

## Discussion

The aim of the current study was to verify whether different WB-HIIT protocols would generate different muscle thickness and functional adaptations in healthy and physically active individuals. Confirming the initial hypothesis, our main findings indicate that both experimental protocols (G30 × 10 and G40 × 20) resulted in similar improvements in all the assessed variables after 6 weeks of intervention.

To our knowledge, studies comparing different training configurations that involve only WB-HIIT protocols are scarce, with most investigations being directed towards comparisons with traditional HIIT, performed on a treadmill (25) or cycle ergometer (26). Therefore, this somehow challenges the comparisons of our findings with others.

The WB-HIIT protocols adopted in our study were sufficient to induce significant hypertrophy for the VL muscle. Our results are in line with previous investigations that reported WB-HIIT as a feasible training approach to increase muscle size. Evangelista et al. (24), for example, reported a significant increase in MT of the VL muscle of health individuals after 6 weeks of a 40 × 20 WB-HIIT protocol. or associated with external load (e.g., kettlebell) (12). Similar to our findings, Moghaddam et al. (12) also failed to demonstrate distinct muscular morphological adaptations (muscle cross sectional area) in recreationally active participants following two different WB-HIIT protocols (10-5-HIIT vs. 20-10-HIIT). Interestingly, the mean relative hypertrophic response was similar between the present study and Moghaddam et al. (12) (10.5% and 9.7%, respectively) when accounting for the values of the whole sample. In general, the positive effects of both WB-HIIT programs herein observed may be explained by the exercises included in the training programs (squat jump and sumo squat). Additionally, the insertion of plyometric exercises (e.g., squat jump) in WB-HIIT routines may produce positive effects in hypertrophy of the lower limb muscles when compared to traditional resistance training (27). High intensity interval training using body weight also involves exercises with high-speed execution and short rest periods combined with stretching-shortening cycles, which favor the recruitment of type 2 muscle fibers, thus promoting muscle hypertrophy (7, 28). From a mechanistic perspective, the positive effects of WB-HIIT on muscle mass outcomes may be explained by the fact that the high tensile stress placed upon the skeletal muscle during HIIT programs have the potential to upregulate cellular mechanisms, specially through the expression of genes and proteins implicated in muscle mass regulation, increase muscle protein synthesis and activate muscle satellite cells (28). Therefore, although high-intensity interval exercise induces a smaller increase in myofibrillar protein synthesis compared to resistance-type exercise (29), both WB-HIIT programs studied by the present investigation may be feasible approaches to be implemented in training programs aiming to promote skeletal muscle hypertrophy.

The improvements associated with VO_2_max through the application of WB-HIIT protocols are already well documented in the literature (30). The percentage increase from baseline reported herein (∼10%) is in accordance with previous investigations [∼7% and ∼16% from McRae et al. (8) and Schaun et al. (25), respectively]. Additionally, the magnitude of the effect sizes for both WB-HIIT protocols observed herein are within the 95% confidence interval of improvement in VO_2_max (0.28–1.23) recently reported in a meta-analytic investigation (7). The absence of distinct effects between WB-HIIT protocols were already described in the study of Maghaddam et al. (13), in which similar increases in VO_2_max were observed when comparing 10-5-HIIT vs. 20-10-HIIT protocols (+9.4% and 8.9%, respectively) after 4 weeks. These findings point out that short-duration protocols with all-out efforts are able to generate relevant improvements in cardiorespiratory fitness in healthy active individuals, with repeated performance of WB-HIIT exercises causing a cardiopulmonary output comparable to traditional endurance training (31). The observed improvements in cardiorespiratory fitness in the present study may be partially explained by increased mitochondrial volume and density, along with elevated plasma and blood volumes resulting from high stroke volume due to low-volume HIIT protocols (6, 32, 33). It is important to acknowledge that larger increases in VO_2_max could be expected if a longer intervention period was afforded by the present study. This statement holds true based on a linear dose-response relationship between increases in cardiorespiratory fitness and the total training time implemented during WB-HIIT programs (7).

The similar improvements in muscular endurance between training protocols were somehow already expected, since a previous investigation (8) has already reported increases in both sit-up and push-up tests (64% and 135%, respectively) in physically active women after just 4 weeks of training. These findings suggest that WB-HIIT protocols, originally designed to improve aerobic fitness, can also improve muscular endurance in healthy individuals (8). Additionally, it seems that the improvement in muscular endurance is dependent on/specific to the exercises adopted in the training routines. Essentially, exercises that involve pushing, pulling, lifting, and jumping will improve the performance of the muscles associated with these movements (24). Therefore, from a practical standpoint, recreationally active individuals aiming to increase their functional parameters may benefit from an HIIT protocol performed exclusively with body weight exercises, regardless of the training session configuration.

The present study is not without limitations. Firstly, we acknowledge that the study lacks a control group, which limits the ability to draw definitive conclusions about the causal effects of WB-HIIT. Future studies should include a control group to strengthen the evidence base and allow for more rigorous comparisons between WB-HIIT and other training modalities. The short duration of the intervention period, a better control of the physical activity levels of the subjects, and the low sample size must be considered as well. Importantly, the fitness tests used were based on field protocols, and therefore, must be viewed with caution. Also, the training volume (total number of repetitions) was not controlled, which should be considered in futures studies. In addition, subjective variables that play a relevant role in exercise adherence (e.g., enjoyment and perceived exertion) should be addressed in future investigations that aim to compare distinct WB-HIIT protocols. Lastly, the recruitment of physical education students limits the generalizability of our findings to other populations, such as older adults or sedentary individuals. We recommend that future research include a more diverse sample of participants to improve the applicability of the findings across different groups.

## Conclusions

Our findings suggest that, regardless of the training configuration, both WB-HIIT programs serve as time-efficient strategies to induce changes in muscle thickness of the vastus lateralis and functional adaptations in healthy and physically active individuals, allowing the exercises to be performed almost anywhere, making them highly accessible to the general population, especially for those who have limited or no access to fitness equipment and facilities. Therefore, we suggest that both training protocols are suitable options for exercise programs designed to promote improvements in general health and physical fitness parameters.

## Data Availability

The raw data supporting the conclusions of this article will be made available by the authors under reasonable request.

## References

[1] Feito Y Heinrich KM Butcher SJ Poston WSC. High-intensity functional training (HIFT): definition and research implications for improved fitness. Sports. (2018) 6(3):76. 10.3390/sports6030076 30087252 PMC6162410

[2] Menz V Marterer N Amin SB Faulhaber M Hansen AB Lawley JS. Functional vs. Running low-volume high-intensity interval training: effects on VO_2_max and muscular endurance. J Sports Sci Med. (2019) 18(3):497–504. 31427872 PMC6683610

[3] Schaun GZ Pinto SS Brasil B Nunes GN Alberton CL. Neuromuscular adaptations to sixteen weeks of whole-body high-intensity interval training compared to ergometer-based interval and continuous training. J Sports Sci. (2019) 37(14):1561–9. 10.1080/02640414.2019.1576255 30724683

[4] Songsorn P Somnarin K Jaitan S Kupradit A. The effect of whole-body high-intensity interval training on heart rate variability in insufficiently active adults. J Exerc Sci Fit. (2022) 20(1):48–53. 10.1016/j.jesf.2021.10.003 34987590 PMC8689198

[5] Scoubeau C Carpentier J Baudry S Faoro V Klass M. Body composition, cardiorespiratory fitness, and neuromuscular adaptations induced by a home-based whole-body high intensity interval training. J Exerc Sci Fit. (2023) 21(2):226–36. 10.1016/j.jesf.2023.02.004 36970125 PMC10034507

[6] Ma JK Scribbans TD Edgett BA Boyd JC Simpson CA Little JP Extremely low-volume, high-intensity interval training improves exercise capacity and increases mitochondrial protein content in human skeletal muscle. Open J Mol Integr Physiol. (2013) 3:202–10. 10.4236/ojmip.2013.34027

[7] Scoubeau C Bonnechère B Cnop M Faoro V Klass M. Effectiveness of whole-body high-intensity interval training on health-related fitness: a systematic review and meta-analysis. Int J Environ Res Public Health. (2022) 19(15):9559. 10.3390/ijerph19159559 35954911 PMC9367756

[8] McRae G Payne A Zelt JG Scribbans TD Jung ME Little JP Extremely low volume, whole-body aerobic-resistance training improves aerobic fitness and muscular endurance in females. Appl Physiol Nutr Metab. (2012) 37(6):1124–31. 10.1139/h2012-093 22994393

[9] Gist NH Freese EC Ryan TE Cureton KJ. Effects of low-volume, high-intensity whole-body calisthenics on army ROTC cadets. Mil Med. (2015) 180(5):492–8. 10.7205/MILMED-D-14-00277 25939101

[10] Poon ET Chan KW Wongpipit W Sun F Wong SH. Acute physiological and perceptual responses to whole-body high-intensity interval training compared with equipment-based interval and continuous training. J Sports Sci Med. (2023) 22(3):532–40. 10.52082/jssm.2023.532 37711706 PMC10499152

[11] Machado AF Baker JS Figueira Junior AJ Bocalini DS. High-intensity interval training using whole-body exercises: training recommendations and methodological overview. Clin Physiol Funct Imaging. (2019) 39(6):378–83. 10.1111/cpf.12433 28471050

[12] Moghaddam M Estrada CA Baghurst T Jacobson BH. Muscular morphological adaptations of two whole-body high intensity interval training configurations. J Sports Med Phys Fitness. (2020) 60(7):985–91. 10.23736/S0022-4707.20.10526-7 32343081

[13] Moghaddam M Estrada CA Muddle TWD Magrini MA Jenkins NDM Jacobson BH. Similar anaerobic and aerobic adaptations after 2 high-intensity interval training configurations: 10:5 s vs. 20:10 s work-to-rest ratio. J Strength Cond Res. (2021) 35(6):1685–92. 10.1519/JSC.0000000000002939 30829982

[14] Matsudo S Araújo T Matsudo V Andrade D Andrade E Oliveira LC Questionário internacional de atividade física (IPAQ): estudo de validade e reprodutibilidade no Brasil. Rev Bras Ativ Fís Saúde. (2001) 6(2):5–18. 10.12820/rbafs.v.6n2p5-18

[15] WHO Guidelines on Physical Activity and Sedentary Behavior. Geneva: World Health Organization (2020).

[16] Abe T DeHoyos DV Pollock ML Garzarella L. Time course for strength and muscle thickness changes following upper and lower body resistance training in men and women. Eur J Appl Physiol. (2000) 81(3):174–80. 10.1007/s004210050027 10638374

[17] Pollock ML Wilmore JH. Exercises on Health and Illness. Evaluation and Prescription for Prevention and Rehabilitation. 2nd ed. MEDSI (1990). p. 754.

[18] Vandana SD Prafull K Singhal A John N John J. An appraisal of flexibility and agility in Indian Basketball, Volleyball and Handball players and its comparison among them. Indian J Clin Anat Physiol. (2021) 8(1):24–9. 10.18231/j.ijcap.2021.006

[19] Santana JDR De Sá VL. Manual ilustrado do teste de aptidão física para o ingresso na polícia militar do estado do espírito santo. Vitória. (2018). [Monografia de Conclusão de Curso- Universidade Federal do Espírito Santo]. p. 5–50.

[20] Schmitz B Pfeifer C Kreitz K Borowski M Faldum A Brand SM. The yo-yo intermittent tests: a systematic review and structured compendium of test results. Front Physiol. (2018) 5(9):870. 10.3389/fphys.2018.00870 PMC6041409 30026706

[21] Wood R. “*All About The Yo-Yo Endurance Test Level 1”* The Complete Guide to the Yo-Yo Test, (2018). Available online at: https://www.theyoyotest.com/yye1.htm (accessed July 17, 2024).

[22] Evangelista AL La Scala Teixeira C Machado AF Pereira PE Rica RL Bocalini DS. Effects of a short-term of whole-body, high-intensity, intermittent training program on morphofunctional parameters. J Bodyw Mov Ther. (2019) 23(3):456–60. 10.1016/j.jbmt.2019.01.013 31563355

[23] Wilke J Kaiser S Niederer D Kalo K Engeroff T Morath C Effects of high-intensity functional circuit training on motor function and sport motivation in healthy, inactive adults. Scand J Med Sci Sports. (2019) 29(1):144–53. 10.1111/sms.13313 30276916

[24] Evangelista AL Brigatto FA De Camargo JB Braz TV Bocalini DS Teixeira CV Effect of a short-term whole-body high-intensity interval training on fitness, morphological, and functional parameters in untrained individuals. J Sports Med Phys Fitness. (2022) 62(9):1153–61. 10.23736/S0022-4707.21.12342-4 34156180

[25] Schaun GZ Pinto SS Silva MR Dolinski DB Alberton CL. Whole-body high-intensity interval training induce similar cardiorespiratory adaptations compared with traditional high-intensity interval training and moderate-intensity continuous training in healthy men. J Strength Cond Res. (2018) 32(10):2730–42. 10.1519/JSC.0000000000002594 29746386

[26] Blackwell J Atherton PJ Smith K Doleman B Williams JP Lund JN The efficacy of unsupervised home-based exercise regimens in comparison to supervised laboratory-based exercise training upon cardio-respiratory health facets. Physiol Rep. (2017) 5(17):e13390. 10.14814/phy2.13390 28912129 PMC5599857

[27] Earp JE Newton RU Cormie P Blazevich AJ. Inhomogeneous quadriceps femoris hypertrophy in response to strength and power training. Med Sci Sports Exerc. (2015) 47(11):2389–97. 10.1249/MSS.0000000000000669 25811947

[28] Callahan MJ Parr EB Hawley JA Camera DM. Can high-intensity interval training promote skeletal muscle anabolism? Sports Med. (2021) 51(3):405–21. 10.1007/s40279-020-01397-3 33512698

[29] Bell KE Séguin C Parise G Baker SK Phillips SM. Day-to-Day changes in muscle protein synthesis in recovery from resistance, aerobic, and high-intensity interval exercise in older men. J Gerontol A Biol Sci Med Sci. (2015) 70(8):1024–9. 10.1093/gerona/glu313 25650305

[30] Machado AF Zovico PVC Leite CDFC Rica RL Barros BM Evangelista AL Adaptaciones morfofuncionales en programas HIIT de peso corporal: una revisión sistemática (Morphofunctional adaptations in bodyweight HIIT programs: systematic review). Retos. (2024) 57:306–17. 10.47197/retos.v57.104727

[31] Wilke J Mohr L. Chronic effects of high-intensity functional training on motor function: a systematic review with multilevel meta-analysis. Sci Rep. (2020) 10(1):21680. 10.1038/s41598-020-78615-5 33303848 PMC7728805

[32] Gibala MJ Little JP van Essen M Wilkin GP Burgomaster Ka Safdar A Short-term sprint interval versus traditional endurance training: similar initial adaptations in human skeletal muscle and exercise performance. J Physiol. (2006) 575(Pt 3):901–11. 10.1113/jphysiol.2006.112094 16825308 PMC1995688

[33] Sperlich B Wallmann-Sperlich B Zinner C Von Stauffenberg V Losert H Holmberg HC. Functional high-intensity circuit training improves body composition, peak oxygen uptake, strength, and alters certain dimensions of quality of life in overweight women. Front Physiol. (2017) 8:172. 10.3389/fphys.2017.00172 28420999 PMC5376588

